# Draft Genome Sequences of *Yersinia frederiksenii*, *Yersinia intermedia*, and *Yersinia kristensenii* Strains from Brazil

**DOI:** 10.1128/genomeA.00780-17

**Published:** 2017-08-10

**Authors:** Priscilla Fernanda Martins Imori, Fábio Campioni, Guojie Cao, George Kastanis, Maria Sanchez Leon, Marc William Allard, Juliana Pfrimer Falcão

**Affiliations:** a Departamento de Análises Clínicas, Toxicológicas e Bromatológicas, Faculdade de Ciências Farmacêuticas de Ribeirão Preto, Universidade de São Paulo, Ribeirão Preto, São Paulo, Brasil; b Division of Microbiology, Office of Regulatory Science, Center for Food Safety and Applied Nutrition, U.S. Food and Drug Administration, College Park, Maryland, USA

## Abstract

*Yersinia enterocolitica-*like strains are usually understudied. In this work, we reported the draft genome sequences of two *Yersinia frederiksenii*, two *Yersinia intermedia*, and two *Yersinia kristensenii* strains isolated from humans, animals, food, and the environment in Brazil. These draft genomes will provide better molecular characterizations of these species.

## GENOME ANNOUNCEMENT

Within the *Yersinia* genus, *Y. frederiksenii*, *Y. intermedia*, and *Y. kristensenii* are often categorized as *Y. enterocolitica*-like species and have been isolated from healthy and sick humans and animals, as well as from food and environmental sources (1–4). These species are usually considered to be nonpathogenic, and for this reason they have been poorly studied (4). However, some studies have reported the isolation of these species in clinical cases from humans and animals (4–7).

In this announcement, we report six draft genome sequences of *Yersinia enterocolitica*-like strains, two *Yersinia frederiksenii*, two *Yersinia intermedia*, and two *Yersinia kristensenii* strains, isolated in Brazil and identified phenotypically according to Schriefer et al. (8). DNA from each strain was extracted according to Campioni et al. (9). Libraries were prepared using 1 ng of genomic DNA with the Nextera XT DNA library preparation kit (Illumina, San Diego, CA). The genomes were then sequenced using the Illumina NextSeq 500 desktop sequencer with the NextSeq 500/500 high-output kit V2 (300 cycles) (Illumina, San Diego, CA) at 2 × 151 cycles according to the manufacturer’s recommendations. *De novo* assemblies were generated from all raw sequence data, and the Illumina reads were assembled with CLC Genomic Workbench version 9.5.2. The contigs for each isolate (draft genomes) were annotated using NCBI’s Prokaryotic Genome Annotation Pipeline (PGAP) (10). The genomes ranged between 4.8 and 5.0 Mb in size for *Yersinia frederiksenii*, between 4.9 and 5.1 Mb in size for *Yersinia intermedia*, and between 4.6 and 4.8 Mb in size for *Yersinia kristensenii*, as described for *Yersinia* spp. (3.7 Mb to greater than 5.0 Mb) (11). The number of contigs per assembly for each isolate ranged between 78 and 145.

The data provided will help better characterize *Yersinia frederiksenii*, *Yersinia intermedia*, and *Yersinia kristensenii* species at the genomic level. A more detailed report of these genomic features will be addressed in a future publication.

### Accession number(s).

These six draft genomes have been deposited in GenBank under the accession numbers NHOF00000000, NHOG00000000, NHOH00000000, NHOI00000000, NHOJ00000000, and NHOK00000000.
